# RECQL: a DNA helicase in breast cancer

**DOI:** 10.18632/oncotarget.5452

**Published:** 2015-09-03

**Authors:** Mohammad R. Akbari, Cezary Cybulski

**Affiliations:** Women’s College Research Institute, Women’s College Hospital, Dalla Lana School of Public Health, University of Toronto, Toronto, ON, Canada

**Keywords:** Chromosome Section, RECQL, breast cancer, risk assessment, treatment

We recently reported that mutations in the *RECQL* gene are associated with an increased risk of breast cancer [1]. We identified recurrent *RECQL* mutations in two founder populations in Poland and Quebec, Canada, and we found that these mutations increase the risk of breast cancer by 5-fold among unselected cases from Poland and by 16-fold among higher-risk cases in Quebec. The association of *RECQL* mutations with breast cancer has been also confirmed in a Chinese population [2], suggesting that *RECQL* mutations are not limited to a specific population.

The RECQ family of DNA helicases has five members, of which RECQL is the smallest and most abundantly expressed transcript in human cells [3]. Phenotypes seen in RECQL-depleted mouse and human cells suggest that RECQL has an important function in maintaining the genome integrity [4]. It seems that RECQL prevents dsDNA breaks by restabilising stalled replication forks, which can result in dsDNA breaks if they are not resolved in a timely manner by RECQL and its other associated proteins [5]. The tumour suppressor function of RECQL and its breast cancer predisposing nature we reported seem to contradict the high expression of the gene seen in cancer cells [6]. But, this paradox could be explained when we consider the role RECQL plays in DNA replication and how highly proliferative cells like cancer cells would probably need it to survive. It is plausible to postulate that the high expression of RECQL in cancer cells is secondary to acquiring a tumor phenotype in these cells; otherwise RECQL is a tumour suppressor in normal cells by maintaining the integrity of the genome. This needs to be investigated in future studies on the role of RECQL in cancer pathogenesis.

Our study observed an odds ratio of 5 for breast cancer developing among unselected Polish patients with *REQCL* mutations, representing a life time risk of about 50%. Although it appears that *RECQL* is a high penetrance breast cancer susceptibility gene, it seems that a small proportion of breast cancer patients (1 in 400) carry a truncating *RECQL* mutation. This mutation frequency is based on studying unselected Polish breast cancer patients and just based on a single recurrent truncating mutation. To get a better estimate of the *RECQL* mutation frequency, we need to study other populations for mutations in the entire coding region of the gene. In addition, we need to find out whether any of the observed *RECQL* missense mutations are pathogenic or not. It is noteworthy to mention that all of the 14 *RECQL* missense mutations identified among the 1,145 sequenced patients in our study had a minor allele frequency lower than 0.5%. Since the possibility of being pathogenic is higher for rare missense mutations compared to more common missense variants, this low frequency suggests that some of these mutations could potentially be pathogenic. However, we need to establish some functional assays to investigate the effect of *RECQL* missense mutations on protein function.

Whatever the frequency is of pathogenic *RECQL* mutations, probably it will not be high enough to justify the screening of breast cancer patients or the general population for mutations in this gene alone. It would make sense, however, to add the *RECQL* gene to breast cancer susceptibility gene panels and screen individuals for mutations in this gene and others. Before we can fully benefit from such a screening though, we still need to do more studies to get a better idea about the life time risk of breast cancer among *RECQL* mutation carriers. Assuming that the life time risk of 50% estimated from our study is consistent with further research, then prophylactic measures such as double mastectomy could be recommended for *RECQL* mutation carriers.

The other potential implication of identifying *RECQL* as a breast cancer susceptibility gene is for the treatment of patients carrying *RECQL* mutations. Since the RECQL protein restarts replication forks reversed by topoisomerase I (TOP1) inhibitors and allows some cancer cells to survive the cytotoxic effect of this class of drugs, it is suggested that the down-regulation of *RECQL* in combination with TOP1 inhibitors could be more effective in treating cancer patients [5]. With the same argument, we can expect that breast cancer patients who are carriers of deleterious *RECQL* mutations might also respond better to TOP1 inhibitors, such as Camptosar and Hycamtin. This is a hypothesis based on what we know from the literature about the function of RECQL and we need to conduct more research with well-designed clinical trials for investigating it.

We identified *RECQL* as a breast cancer susceptibility gene through re-sequencing cases in two founder populations from Poland and Quebec. First we did exome sequencing on 195 cases and found several *RECQL* mutation carriers, and then we confirmed the association with breast cancer by studying about 25,000 more cases and controls. The identification of recurrent founder mutations in both populations in the discovery phase allowed us to investigate further their associations in two large samples of cases and controls, in an efficient and cost-effective manner. This approach of using founder populations is likely to be successful in the search for other rarely mutated cancer susceptibility genes.

## References

[1] Cybulski C (2015). Nat Genet.

[2] Sun J (2015). PLoS Genet.

[3] Puranam KL (1994). J Biol Chem.

[4] Sharma S (2007). Mol Cell Biol.

[5] Berti M (2013). Nat Struct Mol Biol.

[6] Futami K (2008). Cancer Sci.

